# Direct Generation of Neurosphere-Like Cells from Human Dermal Fibroblasts

**DOI:** 10.1371/journal.pone.0021801

**Published:** 2011-07-13

**Authors:** Soon-Tae Lee, Kon Chu, Keun-Hwa Jung, Young-Mi Song, Daejong Jeon, Seung U. Kim, Manho Kim, Sang Kun Lee, Jae-Kyu Roh

**Affiliations:** 1 Department of Neurology, Clinical Research Institute, Seoul National University Hospital, Seoul, South Korea; 2 Program in Neuroscience, Neuroscience Research Institute of SNUMRC, Seoul National University, Seoul, South Korea; 3 Department of Bio and Brain Engineering, Korea Advanced Institute of Science and Technology (KAIST), Daejeon, South Korea; 4 Division of Neurology, Department of Medicine, UBC Hospital, University of British Columbia, Vancouver, Canada; 5 Medical Research Institute, Chungang University School of Medicine, Seoul, South Korea; University of Sao Paulo – USP, Brazil

## Abstract

Neural stem cell (NSC) transplantation replaces damaged brain cells and provides disease-modifying effects in many neurological disorders. However, there has been no efficient way to obtain autologous NSCs in patients. Given that ectopic factors can reprogram somatic cells to be pluripotent, we attempted to generate human NSC-like cells by reprograming human fibroblasts. Fibroblasts were transfected with NSC line-derived cellular extracts and grown in neurosphere culture conditions. The cells were then analyzed for NSC characteristics, including neurosphere formation, gene expression patterns, and ability to differentiate. The obtained induced neurosphere-like cells (iNS), which formed daughter neurospheres after serial passaging, expressed neural stem cell markers, and had demethylated *SOX2* regulatory regions, all characteristics of human NSCs. The iNS had gene expression patterns that were a combination of the patterns of NSCs and fibroblasts, but they could be differentiated to express neuroglial markers and neuronal sodium channels. These results show for the first time that iNS can be directly generated from human fibroblasts. Further studies on their application in neurological diseases are warranted.

## Introduction

Neural stem cell (NSC) transplantation is a promising tool for inducing the regeneration of damaged brain [1]. In addition, NSCs have disease-modifying effects in neurologic diseases, such as anti-inflammation, immune modulation, and neuroprotection [1], [2], [3], [4], [5]. Thus, the production of customized autologous NSCs has been of interest to many researchers seeking a feasible source of cells for cell therapy in neurologic diseases.

Currently, NSCs can be obtained in two ways. The first is by culturing human subventricular zone tissues in biopsied or autopsied specimens [6]. However, doing this for autologous cells is very difficult because of its invasiveness, and the use of allogeneic cells from aborted fetuses is controversial and there is little tissue available. NSCs can also be obtained by the controlled differentiation of allogeneic embryonic stem cell lines (ESCs) or autologous induced pluripotent cells (iPS) [7].

Reprogramming of fibroblasts by transfection with Oct3/4, Sox2, Myc, and Klf4 or Oct4, Sox2, Nanog, and Lin28 results in iPS that resemble ESCs [8], [9], [10]. However, this requires viral integration of *c-myc* into the host genome, which increases the risk of tumorigenicity [11]. Therefore, several modified methods have been developed for transfection, including non-viral plasmid transfection of the factors [12], generation of iPS without *myc* [13], the use of the piggyBac transposon system [14], or the use of proteins to replace viral vectors [15]. Nevertheless, for transplantation in neurologic diseases, ESC or iPS should be differentiated again into neural stem cells (NSCs) or neuroglial cells. This still carries a long-term risk of tumorigenicity due to remnant undifferentiated pluripotent cells [16], [17], [18], [19].

Recently, the direct generation of neurons or cardiomyocytes from mouse fibroblast has been reported, suggesting that it is possible to induce linage-committed cells without achieving pluripotency [20], [21]. In addition, transfection of fibroblasts with cellular protein extracts from mouse ESCs have been reported to induce fibroblasts to become pluripotent stem cells, suggesting that the cellular extracts can replace the viral reprogramming factors [15], [22]. Previous studies suggest that various cell extracts can be used for the donor cell-like reprogramming of recipient cells [23], [24]. Thus, we hypothesized that fibroblasts can be induced to become NSC-like cells by introducing them with cell extracts derived from NSCs. Here, we show that NSC lines (NSCLs) in place of NSCs, can be used for the large-scale production of cell extracts that are able to induce fibroblasts to become neurosphere-like cells (iNS).

## Results

### Generation of iNS

**Figure 1:**
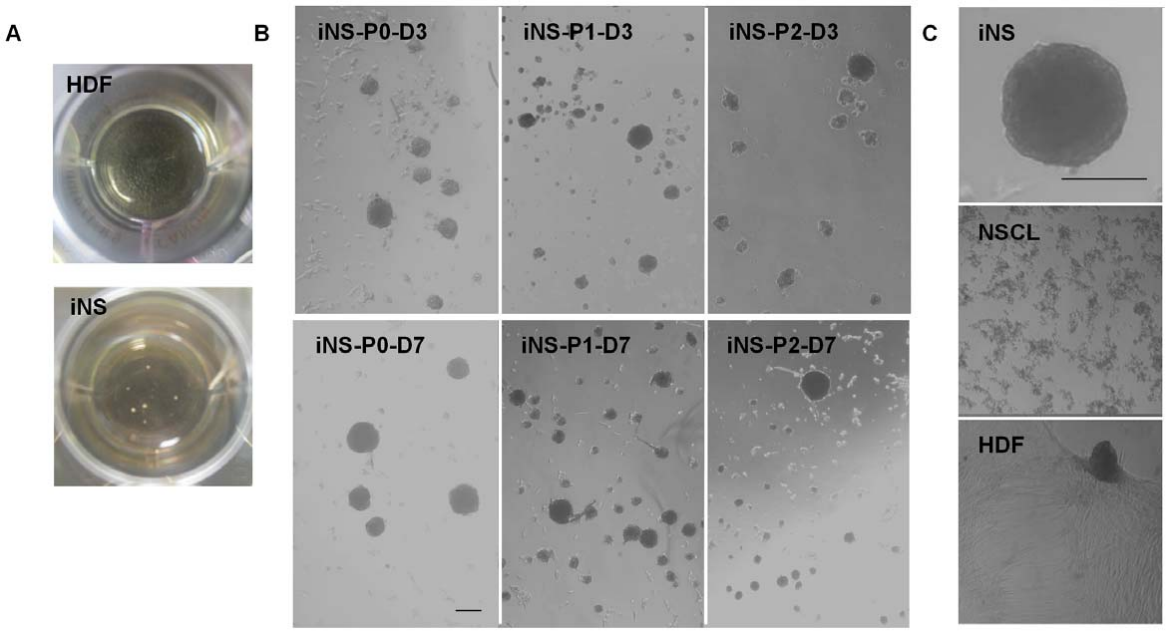
Generation of neurosphere-like cells from HDF. HDF grown in neurosphere culture conditions did not generate neurospheres, whereas sphere formation was detected in iNS cultures (A). Serial passaging of primary iNS (iNS-P0) produced secondary (iNS-P1) and tertiary (iNS-P2) spheres (B). Cultures of NSCL or HDF in neurosphere culture conditions did not produced spheres, and high-density cultures of HDF resulted only in clumping of the cells (C). Bar  = 100 µm.

Between 1.0 and 1.6×10^5^ cells were necessary to generate 1 µL of NSCL extract, and 230 ng/ml was the most effective concentration of SLO for transfection (Figure S1). Higher concentrations induced cell death and did not improve efficiency. Using this concentration of SLO, HDF were transfected with NSCL extracts. When the cells were grown in neurosphere medium for 7 days, they formed spheres after 2–3 days (Figure 1A and Figure S1). Culture of 1.1×10^5^ HDF produced on average 16.5±5.1 spheres. The mean size of spheres was 77±22 µm (n = 120), which is smaller than the reported size of cultured human neurospheres [25]. However, culturing the cells in normal proliferative medium (DMEM+10% FBS) or culturing HDF transfected with HDF extracts in neurosphere medium did not result in sphere formation (Figure S2).

To identify components of NSCL extracts that may be responsible for sphere formation, we also examined the effect of heat-denatured or RNase-treated NSCL extracts. Heat-denatured NSCL or HDF extracts caused cell death and did not result in sphere formation. RNase-treated NSCL extracts resulted in the formation of a few spheres, although there were fewer and smaller than in cells transfected with native NSCL extracts (Figure S3), suggesting that both proteinaceous and RNA components participate in the sphere induction. Time-lapse photography of sphere formation is shown in the video S1.

### Characterization of iNS

We next examined whether iNS can form secondary and tertiary spheres. Primary spheres were serially passaged every 7 days. We observed the formation of secondary (iNS-P1) and tertiary sphere (iNS-P2) (Figure 1B). When labeled with BrdU, cells in the spheres showed evidence of proliferation (Figure S4). However, the growth of iNS was slow so that there was substantial cell loss, preventing the passaging of spheres for more than 3 passages. Growing 9.5×10^4^ iNS-P1 cells for 7 days, followed by passaging resulted in 8.4×10^4^ iNS-P2 cells, suggesting a similar cell number due to the combined effects of cell loss during passaging and subsequent cell proliferation. No clonal neurospheres were observed when single cells were micropipetted into separate 96 wells of neurosphere medium. Although short- or long-term culture of NSCL or HDF in neurosphere medium did not produce spheres, HDF cultured at a high cellular density resulted in the formation of cell clumps that was morphologically different from spheres (Figure 1C). When we used NSCL extract derived from commercial ReN human NSCs for the iNS induction, we also observed sphere formation (Figure S5).

**Figure 2:**
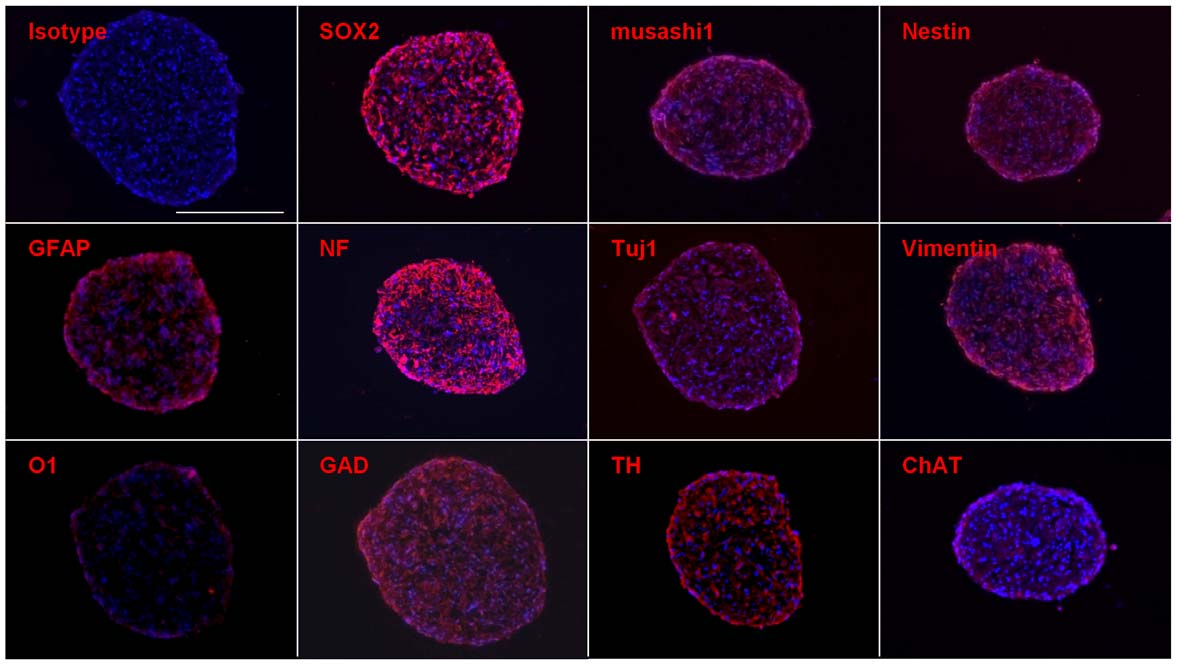
Immunocytochemistry of iNS. iNS-P0 expressed neural stem cell markers, including SOX2 and musashi1, as well as neuroglial markers including nestin, GFAP, neurofilament (NF), Tuj1, vimentin, anti-human Tuj-1, neurofilament, glutamic acid decarboxylase (GAD), tyrosine hydroxylase (TH), and a low level of ChAT. Bar  = 100 µm.

To determine if the iNS have neurosphere-like characteristics, we examined them for the presence of various neural stem/progenitor or neuronal markers by immunocytochemistry. We found that iNS express SOX2 and musashi1, which are expressed at high level in NSC [26], [27], as well as nestin, GFAP, neurofilament, Tuj1, vimentin, glutamic acid decarboxylase, tyrosine hydroxylase, and a low level of choline acetyltransferase (Figure 2).

**Figure 3:**
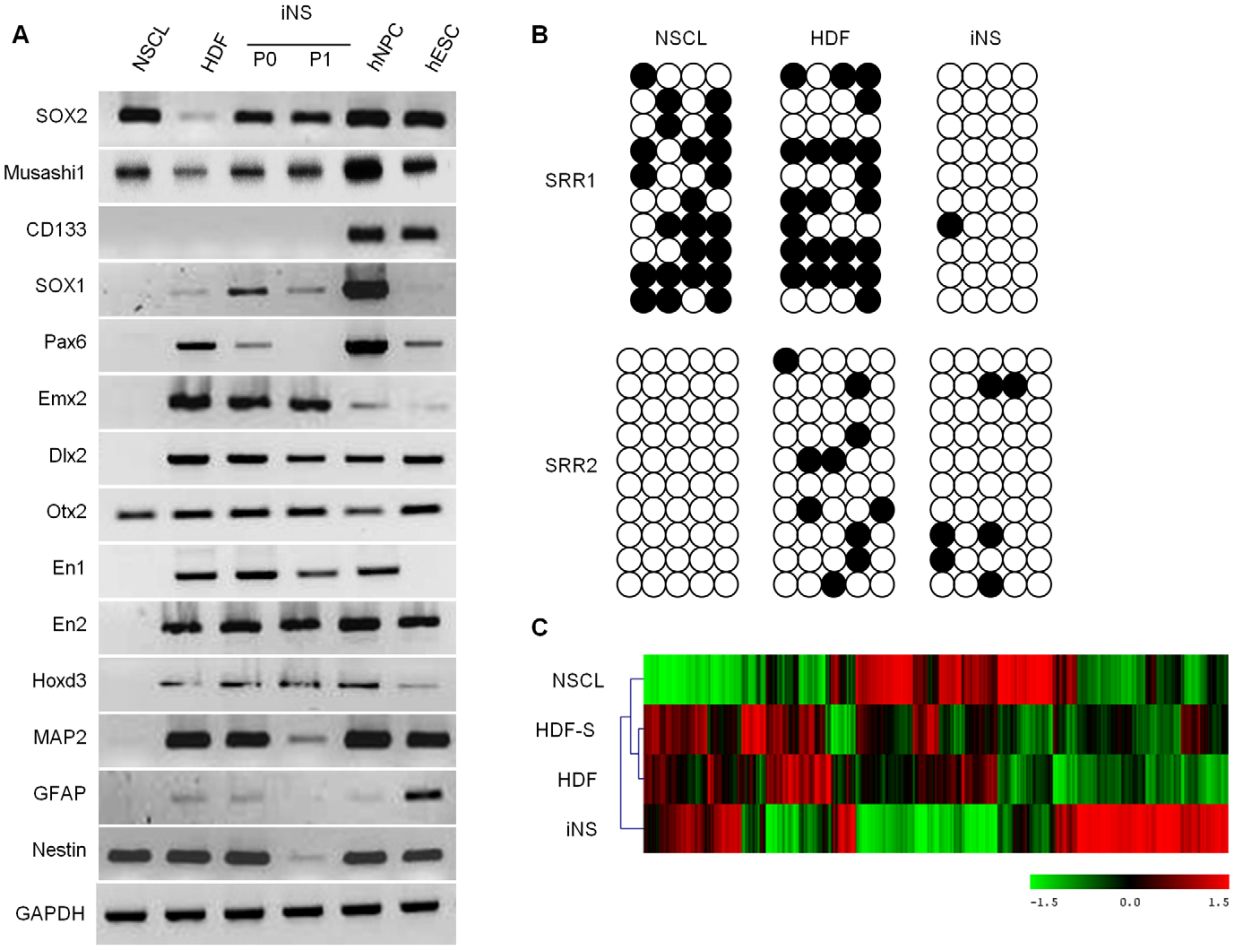
Gene expression by iNS. RT-PCR of iNS-P0, NSCL, and hNPC showed that they commonly express high levels of *SOX2* and *musashi1* compared to HDF (A). *CD133* was expressed in human NPC and ESC. HDF, iNS-P0, and hNPC shared some common markers expressed during central nervous system development. iNS-P1 showed reduced or no expression of *Pax6*, *nestin*, *GAFP*, and *MAP2* compared to iNS-P0. Bisulfide methylation analysis of the two *SOX2* regulatory regions (*SRR1* and *SRR2*) indicated demethylation of both *SRR1* and *SRR2* in iNS-P0, whereas NSCL showed demethylation of *SRR2* only and HDF showed substantial methylation of both *SRR1* and *SRR2* (B). Microarray analysis indicated a different pattern of gene expression in iNS-P0 than in NSCL and HDF (C). Expression patterns were similar for conventionally cultured HDF and HDF cultured in neurosphere medium (HDF-S).

To further characterize iNS, we used RT-PCR to measure the mRNA levels of several genes increased in NSCs or developing brain in primary (P0) and secondary (P1) iNS. As a positive control, we included complimentary DNA from non-immortalized primary human neurosphere cells (hNPC, human neural progenitor cells) cultured from human fetal subventricular zone tissue. We found that iNS, NSCL, and hNPC express higher levels of *SOX2* and *musashi-1* compared to HDF, suggesting that the iNS have neurosphere-like characteristics (Figure 3A). The iNS-P0, hNPC, and HDF commonly expressed other developmental genes, including *Pax6*, *Emx2*, *Dlx2*, *Otx2*, *En1*, *En2*, *Hoxd3*, *MAP2*, *GFAP*, and *nestin*. In addition, expression was lower in iNS-P1 than in iNS-P0 for *SOX1*, *Pax6*, *MAP2*, *GFAP*, and *nestin*.

*SOX2* has two regulatory regions, *SRR1* and *SRR2* [28]. DNA methylation analysis indicated substantial demethylations of *SRR1* and *SRR2* in iNS at P0 (Figure 3B). NSCL had substantial methylation of *SRR1* and demethylation of *SRR2*. HDF had demethylation of both regions. Gene expression microarray patterns were distinct for iNS, NSCL, and HDF, whereas non-reprogrammed HDF cultured in neurosphere medium had a similar gene expression pattern as HDF cultured in DMEM supplemented with 10% FBS (Figure 3C).

PCR confirmed that, although NSCL are generated by transfection with *v-myc* [29], iNS were not contaminated by the *v-myc* gene (Figure S6). Also, iNS had normal chromosome numbers and characteristics (Figure S7). Finally, the short tandem repeats in iNS were identical to those of HDF (Figure S8).

### Differentiation of iNS

**Figure 4:**
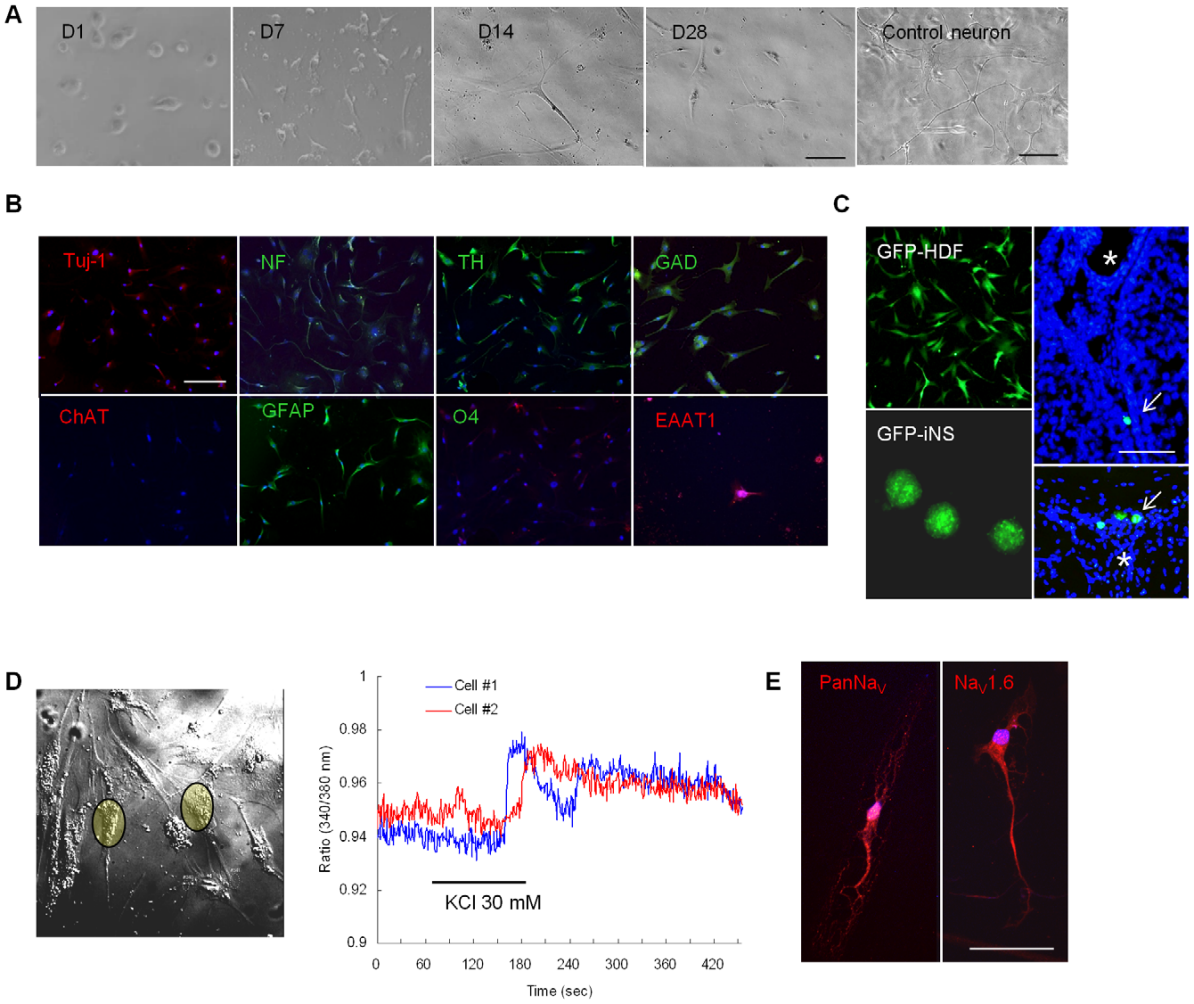
Differentiation of iNS. iNS cultured in differentiation medium spread cytoplasmic processes resembling neurites (A). Mouse neuronal cells were cultured from embryonic hippocampus and presented as a control. Immunocytochemistry showed that the differentiated cells expressed Tuj-1, neurofilament (NF), tyrosine hydroxylase (TH), GAD, GFAP, a low level of O4, and EAAT1 (B), but no choline acetyltransferase (ChAT). GFP-expressing iNS was induced from GFP-expressing HDF and transplanted into the lateral ventricle of neonatal mice at P3 (C). A few cells were integrated into the subventricular zone at 4 days after the transplanation, however, they were disappeared in 2 or 4 weeks after the transplantation even though cyclosporin injection (C). * indicates the lateral ventricle. Ca^2+^ imaging showed that the differentiated cells showed a slow inward Ca^2+^ influx (D). In cultures that were more enriched to induce neuronal differentiation, however, some differentiated cells expressed neuronal sodium channel markers, including PanNav and Nav1.6 (E). Bar  = 50 µm.

When cultured in the differentiation medium (serum- and mitogen-free medium), iNS spontaneously differentiated to have elongated cytoplasmic process that resembled neurites (Figure 4A). Immunostaining showed that differentiated iNS diffusely expressed Tuj-1, neurofilaments, TH, GAD, GFAP, EAAT1, and very low levels of O4 (less than 20% of the cells), suggesting differentiation toward to both neuronal and glial cells (Figure 4B). We generated GFP-expressing iNS by reprogramming HDF labled with GFP-lentivirus (Figure 4C). After intraventricular transplantation of GFP-iNS in the brain of neonatal mice, iNS were observed in the subventricular zone at 4 days after the transplantation. However, no iNS was observed when examined 2 or 4 weeks after the transplantaiton, although the mice were injected with cyclosporin (n = 8 mice). Instead, we checked whether the differentiated cells from iNS have electrophysiologic characteristics of neuron. Intracellular Ca^2+^ imaging from 3000 cells (Figure 4D) indicated that they all showed a slow inward Ca^2+^ in response to KCl. Nervertheless, when iNS were treated under a defined differentiation conditions by adding insulin, transferrin, selenite, and progesterone to the culture, they showed strong expression of the sodium channels Pan Na_v_ and Na_v_1.6 (Figure 4E).

## Discussion

In this study we generated iNS, which have neurosphere-like characteristics, by reprogramming human fibroblasts with cell extracts from NSCLs. This method avoids the need for viral vectors, which can cause genetic instability and may be tumorigenic, and it provides a ready source for human autologous neural progenitor cells. These iNS may be useful for transplantation in disease models and they may have clinical application. However, it is troubling that the growth of iNS was slow, preventing the passaging of spheres for more than 3 passages and the generation of clonally-derived neurospheres. In addition, iNS failed to integrate into the developing brains. Accordingly, we defined iNS as neurosphere-“like” cells underwent incomplete reprogramming. Nevertheless, because NSCLs can be rapidly expanded in vitro, they can provide sufficient source material for the repeated reprogramming of fibroblasts to generate sufficient amounts of iNS-P0 or iNS-P1 cells. Further investigations are necessary to develop modified methods that can provide fully reprogrammed iNS.

ESC-derived extracts allow protein-based cell reprograming of dermal fibroblasts to generate iPS that can differentiate into 3-germ layers *in vitro*, form teratomas, and can be used to generate chimeric mice [15]. Although the key proteinaceous component of the NSCL extracts remains unknown, cell extract-based reprogramming is an effective alternative to using nuclear transfer or cell fusion to insert the reprograming machinery in the donor cytoplasm into the recipient cells [30]. Thus, while further studies regarding the mechanism how NSCL extracts reprogrammed fibroblast is essential, the practical aspect of iNS is warranted enough.

Although the iNS developed here were reprogrammed, they did not have all of the characteristics of NSCs obtained from human subventricular zone tissues. For example, some of the expressed genes were also expressed by HDF, and the iNS had a different gene expression pattern than the donor NSC line. In addition, we could not generate clonally-derived iNS. This might be due to a remaining epigenetic pattern, mRNA, and protein from HDF along with the fact that, unlike *v-myc*-transfected NSCL, iNS are expanded in a sphere-forming and non-adherent culture condition. Indeed, previous studies have shown that iPS have epigenetic memory of the tissue of origin that influences their differentiation [31]. In addition, after the ectopic delivery of reprogramming factors, partially reprogrammed intermediate cells remain, and the selection of fully reprogrammed cells is necessary even for the generation of iPS [15], [32], [33]. The iPS' epigenetic memory of donor tissue can be reset by serial reprogramming or by chromatin-modifying drugs [31]. Although iNS were maintained only for limited passages, repeated generations of iNS can provide sufficient amount of iNS-P0 cells for therapeutic purpose. While iNS failed to form clonal neurospheres, density dependent growth is not uncommon in primary NPCs [34]. Thus, we are now trying to refine the methods for reprograming and subsequent selection of fully transformed iNS.

Although the iNS generated here did not have all of the characteristics of NSCs, they express higher levels of NSC markers, including *SOX2* and *musashi1*, compared to HDF, form neurospheres, have typical patterns of *SRR1* and *SRR2* demethylation, and can be differentiated to express sodium channels. Sox2 is one of the earliest transcription factors expressed in the developing central nervous system and plays a critical role in maintaining the pluripotency of stem cells [26]. Musashi1 is an evolutionally conserved marker for central nervous system progenitor cells [27]. Demethylation of both *SRR1* and *SRR2* indicates that the corresponding cells are neural stem cells, as only *SRR1* is highly methylated in astrocytic cells and both *SRR1* and *SRR2* are methylated in neural cells [28]. Thus, iNS appear to have undergone substantial reprogramming to become neural stem cell-like.

NSC transplantation is a promising tool for regenerating damaged brain tissue in many neurological disorders [1]. Researchers and clinicians are therefore seeking a safe and efficient source of customized NSCs. The ability to generate iNS from autologous fibroblasts is an important advance, and further studies of their characterization and application in neurologic disease models are warranted.

## Materials and Methods

### Cell culture and preparation of cell extracts

The study was approved by the institutional review board of Seoul National University Hospital. We obtained written informed consent from all participants. Human dermal fibroblasts (HDF) were obtained from skin tissue remaining after surgery and were cultured as previously described [9]. Two human NSCLs were used in this study. In most of the experiments, we used HB1.F3 cells, which were derived from human fetal brain and were immortalized with the *v-myc* oncogene [29], [35]. We also used ReNcell VM human neural progenitor cells (Chemicon, Billerica, MA, USA), which were also immortalized with the *v-myc* gene [36]. All NSCLs were maintained in Dulbecco's modified Eagle's medium (DMEM; Invitrogen, Carlsbad, CA, USA) + 10% fetal bovine serum (FBS; Invitrogen).

NSCL extracts were prepared as described previously [15], [22], [37], although with minor modifications. Briefly, harvested NSCLs were washed with cell lysis buffer (100 mM HEPES, pH 8.2, 50 mM NaCl, 5 mM MgCl_2_, 1 mM dithiothreitol, and protease inhibitors; Invitrogen), collected by centrifugation (400 x g for 5 min), resuspended with lysis buffer, and incubated for 40 min on ice. Cells were sonicated on ice using a 3-mm-diameter probe until nuclei were lysed as determined by microscopic examination. Cell lysates were subjected to centrifugation at 1500 x g for 15 min, and supernatants were collected. The protein concentration of the cell lysate was adjusted to 30 mg/ml (Bradford method) by adding buffer, and stored at −80°C. To denature the protein in the NSCL extracts, the extracts were heated for 5 min at 95°C. To remove RNA in the NSCL extracts, the extracts were incubated with 1 U/mL RNase (Ambion, Applied Biosystems, Foster City, CA, USA) for 1 h at 37°C, and the reaction was stopped by the addition of 10 U/mL RNase inhibitor (Ambion).

### Induction of iNS and culture

Streptolysin-O (SLO)-mediated cell permeabilization of HDF and transfection with NSCL extracts were performed as previously described [15], [22], [37], although with minor modifications. Cultured fibroblasts were washed and suspended in Hank's Buffered Salt Solution (1.1×10^5^ cells in 100 µL; Sigma-Aldrich, St. Louis, MO, USA), incubated in a water bath at 37°C for 2 min, and then mixed with freshly prepared SLO (final concentration, 230–800 ng/ml; Sigma-Aldrich). The cell suspensions were incubated in a water bath for 50 min at 37°C with occasional agitation. Permeabilized cell suspensions were collected and diluted with cold Hank's Buffered Salt Solution to remove SLO and then centrifuged at 400 x g for 5 min. After removing supernatants, cell pellets were mixed with 100 µL of NSCL extract supplemented with ATP-regenerating factors (1 mM ATP, 10 mM creatine phosphate, 25 µg/ml creatine kinase, and 100 mM dNTP; Sigma-Aldrich) and then were incubated in a water bath for 1 h at 37°C with shaking. To assess the extent of cell permeabilization by SLO, cells were incubated with Texas red-conjugated dextran (50 µg/mL; Invitrogen) in place of NSCL extract. The transfected cells were plated in 24-well plate (1.1×10^5^ cells per well) with resealing medium (DMEM containing 10% FBS and 2 mM CaCl_2_) and incubated for 2 h. To induce sphere formation, the resealing medium was removed, and the cells were cultured in neurosphere medium [DMEM/F12 medium supplemented with 1% v/v B27, 20 ng/ml fibroblast growth factor-2 (FGF-2), 10 ng/ml leukemia inhibitory factor (LIF) {or 20 ng/ml epidermal growth factor (EGF)}, 8 µg/ml heparin, and 1% v/v penicillin/streptomycin; Invitrogen] in a humidified incubator at 37°C and 5% CO_2_. Half of the medium was replaced every 3 days. Cell spheres grown for 7 to 10 days were passaged by dissociation with 0.05% trypsin/EDTA (Invitrogen). The cells were reseeded in fresh medium at a density of 200,000 cells/well in 24-well plates. For 5-bromo-2-deoxyuridine (BrdU) labeling, the spheres were cultured in neurosphere medium containing 10 µM of BrdU (Sigma-Aldrich) for 3 days.

### Differentiation of iNS

For spontaneous differentiation of iNS, a single-cell suspension of iNS was plated onto 24-well chamber slides coated with 10 µg/ml poly-L-lysine (Sigma-Aldrich) in serum- and mitogen-free DMEM/F12 + B27 medium. For a defined differentiation of iNS into neuronal lineage cells, the dissociated cells were cultured in a differentiation medium [DMEM/F12, 25 µg/ml insulin, 50 µg/ml human transferring, 30 nM sodium selenite, 20 nM progesterone, 100 nM putrescine, and penicillin/streptomycin; all chemicals from Sigma-Aldrich]. Hippocampal neurons were cultured from mouse embryonic hippocampus as previously described [38].

### Immunocytochemistry

Spheres were fixed in 4% paraformaldehyde for 5 minutes, and then cryosectioned into 14- µm sections. Differentiated cells were fixed with 4% paraformaldehyde for 40 min. Spheres and cells were incubated for 2 h with blocking buffer (PBS containing 2% horse serum, 1% bovine serum albumin, and 0.1% Triton X-100) and then overnight at 4°C with either anti-human Tuj-1 (Chemicon), neurofilament (Chemicon), glutamic acid decarboxylase (GAD, Chemicon), tyrosine hydroxylase (TH, Chemicon), choline acetyltransferase (Chemicon), glial fibrillary acidic protein (GFAP; Chemicon), O1 (Chemicon), EAAT1 (Chemicon), BrdU (Chemicon), PanNa_v_ (Sigma-Aldrich), or Na_v_1.6 antibodies (Abcam, Cambridge, MA, USA), followed by 1 h at room temperature with fluorescein isothiocyanate- or Cy3-conjugated anti-rabbit IgG antibodies (Jackson Immunoresearch, West Grove, PA, USA). Nuclei were counterstained with 4′,6-diamidino-2-phenylindole (Vector Laboratories, Burlingame, CA, USA).

### Real-Time PCR

Total RNA, isolated by using Trizol (Invitrogen), was reverse-transcribed using a First-strand cDNA Synthesis Kit (Roche, Basel, Switzerland). Complimentary DNA from primary human neurosphere cells isolated from the fetal subventricular zone were kindly provided by Dr. Stefano Pluchino (IRCCS San Raffaele, Milan, Italy; currently at Cambridge University, UK). PCR was carried out for 25 cycles of 95°C for 40 s, 58°C for 40 s, and 72°C for 40 s using the primer sets shown in Table S1, as described previously [29], [39].

### Bisulfide methylation assay

A CpGenome DNA Modification Kit (Chemicon) was used to detect methylated cytosine residues on *SRR1* and *SRR2* according to the manufacturer's protocol. Bisulfate-modified DNA was amplified by PCR using as previously described [28], using the primer sets described in Table S1. PCR products were gel-purified and cloned into bacteria using a TOPO TA cloning kit (Invitrogen). Ten clones for each ligation were randomly picked and sequenced on an ABI 310 sequencer (Applied Biosystems).

### Chromosome and short tandem repeat analysis

Short tandem repeats were analyzed with a Powerplex 16 system (Promega, Madison, WI, USA) run in an ABI PRISM 3100 Genetic Analyzer (Applied Biosystems) and visualized with Gene Mapper v3.5 (Applied Biosystems). Chromosomal G-band analysis was performed by MGMED Co. (Seoul, South Korea).

### Microarray analysis

RNA was isolated using Trizol (Invitrogen) according to the manufacturer's instructions, and was labeled and hybridized to an Affimetrix GeneChip human gene 1.0 ST array (Affimetrix, Santa Clara, CA, USA) as described according to the manufacturer's instructions. Data were analyzed using Expression Console software version 1.1 (Affimetrix), in which gene expression ratios were normalized by Robust Multichip Analysis according to the manufacturer's protocol. Differentially expressed genes (>2-fold change in any group) were visualized as a heatmap according to Z-scores. All data is MIAME compliant and that the raw data has been deposited in GEO database (accession number: GSE27667).

### Transplantation

To generate GFP-expressing iNS, HDF were infected with lenti-GFP (green fluorescent protein) virus (10^7^ viral particles/mL medium with adding polybrene) for 2 days and expanded. The GFP-expressing HDF was induced into iNS (GFP-expressing iNS) as previously described method. In cryoanesthetized postnatal day 0 (P0) C57BL/6 mice (Central Lab. Animals Inc., South Korea), 2 µl containing 8×10^4^ iNS dissociated in PBS were injected into the lateral ventricle using a 30-guage Hamilton syringe as previously described [40]. All transplant recipients received daily cyclosporin 10 mg/kg given intraperitoneally (Sigma-Aldrich) beginning on the day of transplant. After 4, 14, or 28 days after the transplantation, mice were deeply anesthetized and perfused through the heart with 10 ml of cold saline and 10 ml of 4% paraformaldehyde in 0.1 M PBS. Sections (20 µm thickness) were stained with DAPI. The animal experiment was approved by Institutional Animal Care and Use Committee of Seoul National University Hospital, which was accredited by the Association for the Assessment and Accreditation of Laboratory Animal Care International.

### Intracellular Ca^2+^ imaging

Intracellular Ca^2+^ was visualized with fura-2 acetoxymethyl ester as previously described [41]. Following incubation with fura-2 acetoxymethyl ester (Invitrogen) for 60 min, neurons were washed three times with HEPES-buffered solution (150 mM NaCl, 5 mM KCl, 1 mM MgCl_2_, 2 mM CaCl_2_, 10 mM HEPES, 10 mM glucose, pH adjusted to 7.4 with NaOH) and exposed to 30 mM KCl, after which [Ca^2+^]_i_ was monitored for 4 to 5 min by measuring the absorbance at 340 and 380 nm.

### Live cell imaging

Live cell imaging was performed using an IX81 time-lapse microscope (Olympus, Center Valley, PA, USA) in conjunction with a DP30BW digital camera (Olympus). Images were taken every 10 min for 72 h. Data were analyzed using MetaMorph, version 7.5.6.0 (Molecular Devices, Sunnyvale, CA, USA).

## Supporting Information

Figure S1 **Determination of the optimal SLO concentration.** Serial concentrations of SLO (0 to 800 ng/ml) were used to introduce HDF with cy3-dextran. A concentration of 230 ng/ml was effective, whereas higher concentrations caused modest cell death.
(TIF)

Figure S2 **Determination of optimal culture conditions.** HDF were transfected with extracts either from HDF or NSCL and were cultured in various media for 7 to 10 days. When cultured in neurosphere medium, HDF transfected with extracts from HDF did not form spheres, but HDF transfected with extracts from NSCL formed spheres.
(TIF)

Figure S3 **Transfection of HDF with heat- or RNase-treated extracts.** When HDF were transfected with either heat-treated NSCL extracts, there was extensive cell death, and when transfected with RNase-treated extracts, they formed smaller spheres. This suggested that mainly protein, and possibly an adjuvant RNA component of the extracts, was responsible for the induction of iNS.
(TIF)

Figure S4 **BrdU labeling of iNS.** Primary iNS were cultured in neurosphere culture containing BrdU for 3 days. Nuclei were counterstained with diamidino-2-phenylindole (DAPI). A few BrdU-positive cells were detected, indicating proliferation of iNS.
(TIF)

Figure S5 **iNS generated by NSCL extracts derived from ReN cells.** NSCL extracts derived from ReN cells could induce fibroblasts into neurospheres.
(TIF)

Figure S6 **Genomic DNA PCR for** ***v-myc*** **.** NSCL were transfected with the *v-myc* oncogene using a retrovirus. PCR indicated that only NSCL had the *v-myc* gene. iNS were not contaminated with the gene.
(TIF)

Figure S7 **Chromosomal analysis of iNS.** iNS have a normal chromosome pattern (46XY).
(TIF)

Figure S8 **Analysis for short tandem repeats.** HDF and iNS have the same patterns of short tandem repeats, whereas the pattern for NSCLs was different. This result confirmed that iNS were derived from HDF, not from NSCLs.
(TIF)

Table S1 **Primers used in RT-PCR, PCR, and bisulfide sequencing.**
(DOCX)

Video S1 **Live cell imaging showing the generation of iNS.** Images were taken every 10 min for 72 h.
(WMV)
