# Correction: Direct Generation of Neurosphere-Like Cells from Human Dermal Fibroblasts

**DOI:** 10.1371/annotation/dba2bb39-93a5-4140-8ced-2b63c36379ba

**Published:** 2012-08-29

**Authors:** Soon-Tae Lee, Kon Chu, Keun-Hwa Jung, Young-Mi Song, Daejong Jeon, Seung U. Kim, Manho Kim, Sang Kun Lee, Jae-Kyu Roh

The funding statement was incorrect. The correct statement is: This study was supported by Research Grants from the Korea Healthcare Technology R&D Project, Ministry for Health, Welfare & Family Affairs, Republic of Korea (A100548). JKR was supported by a grant (SC4120) from Stem Cell Research Center of the 21st Century Frontier Research Program funded by the Ministry of Education, Science and Technology, South Korea, and Seoul National University Hospital Research Funds (0320090200, 0420080160). SKL was supported by Seoul National University Hospital Research Funds (0320090160, 0320110160, 0420100200, 0420080620). KC was supported by Seoul National University Hospital Research Funds (0320110360, 0420090280). The funders had no role in study design, data collection and analysis, decision to publish, or preparation of the manuscript. 

