# Supplementary figures and images for: Direct Generation of Neurosphere-Like Cells from Human Dermal Fibroblasts
Source: PLoS One. 2011 Jul 13;6(7):e21801. doi: 10.1371/journal.pone.0021801 (PMC3135606; doi:10.1371/journal.pone.0021801)

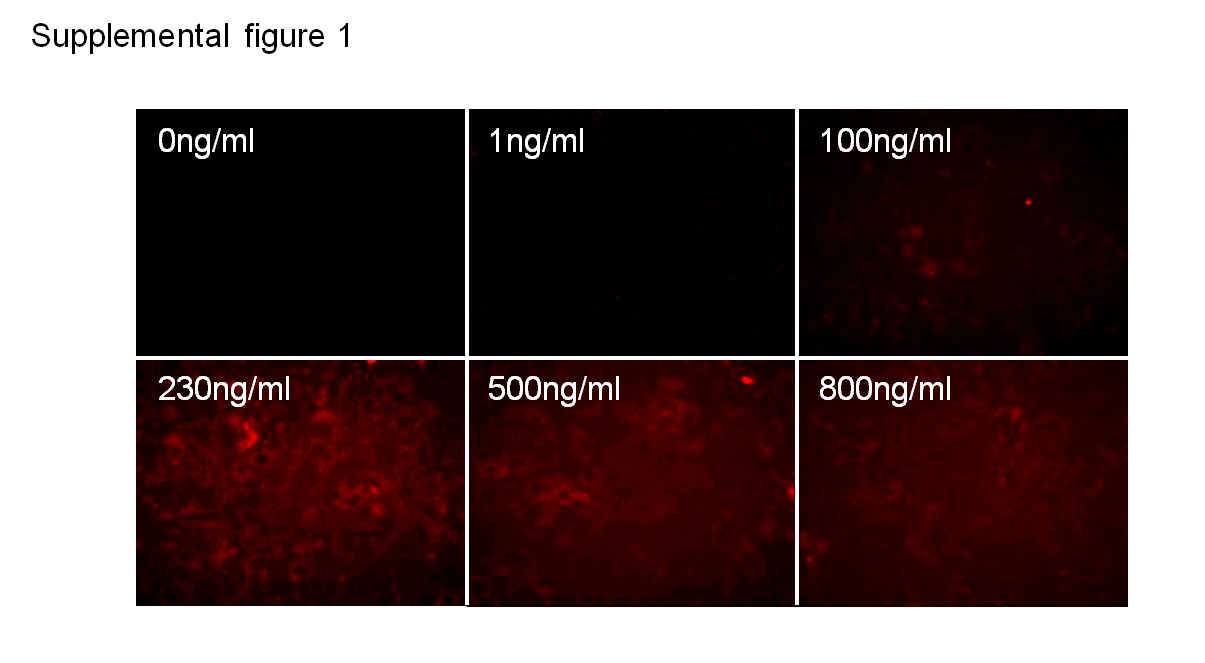

Supplement: Figure S1 — Determination of the optimal SLO concentration. Serial concentrations of SLO (0 to 800 ng/ml) were used to introduce HDF with cy3-dextran. A concentration of 230 ng/ml was effective, whereas higher concentrations caused modest cell death. (TIF) [file pone.0021801.s001.tif]

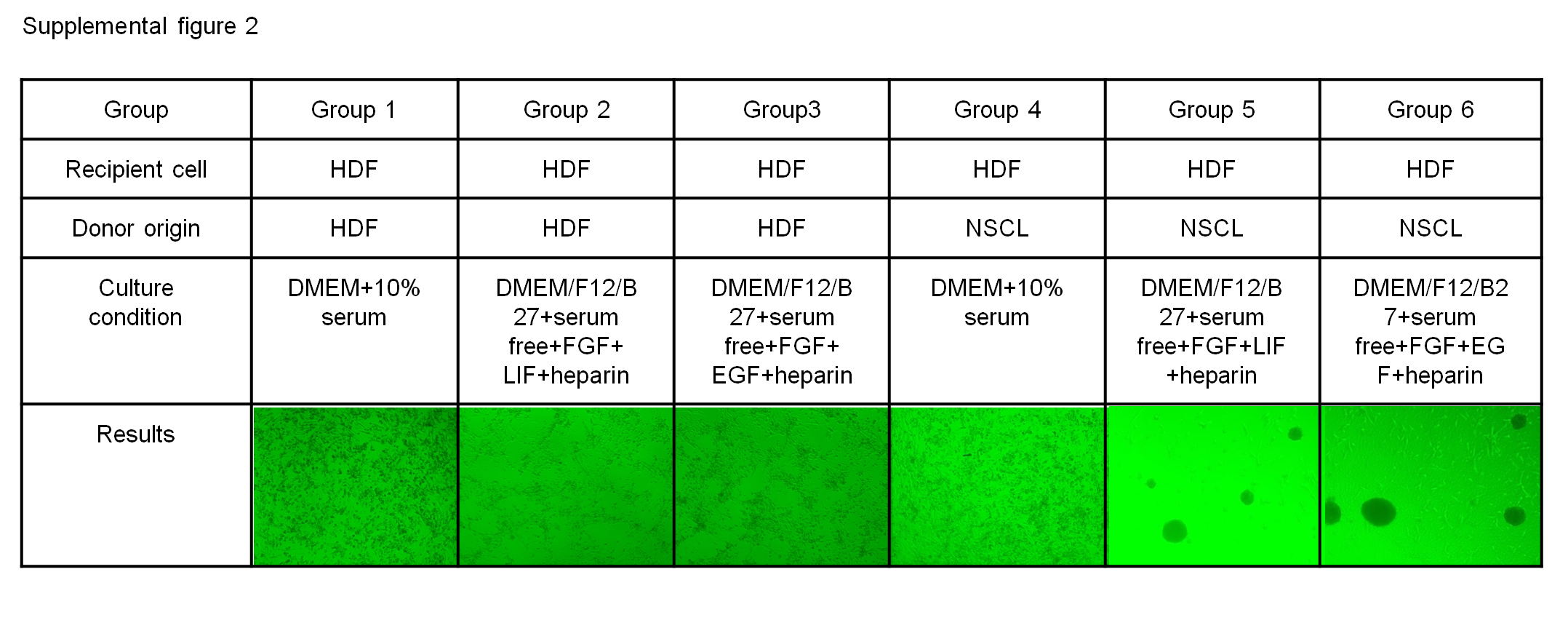

Supplement: Figure S2 — Determination of optimal culture conditions. HDF were transfected with extracts either from HDF or NSCL and were cultured in various media for 7 to 10 days. When cultured in neurosphere medium, HDF transfected with extracts from HDF did not form spheres, but HDF transfected with extracts from NSCL formed spheres. (TIF) [file pone.0021801.s002.tif]

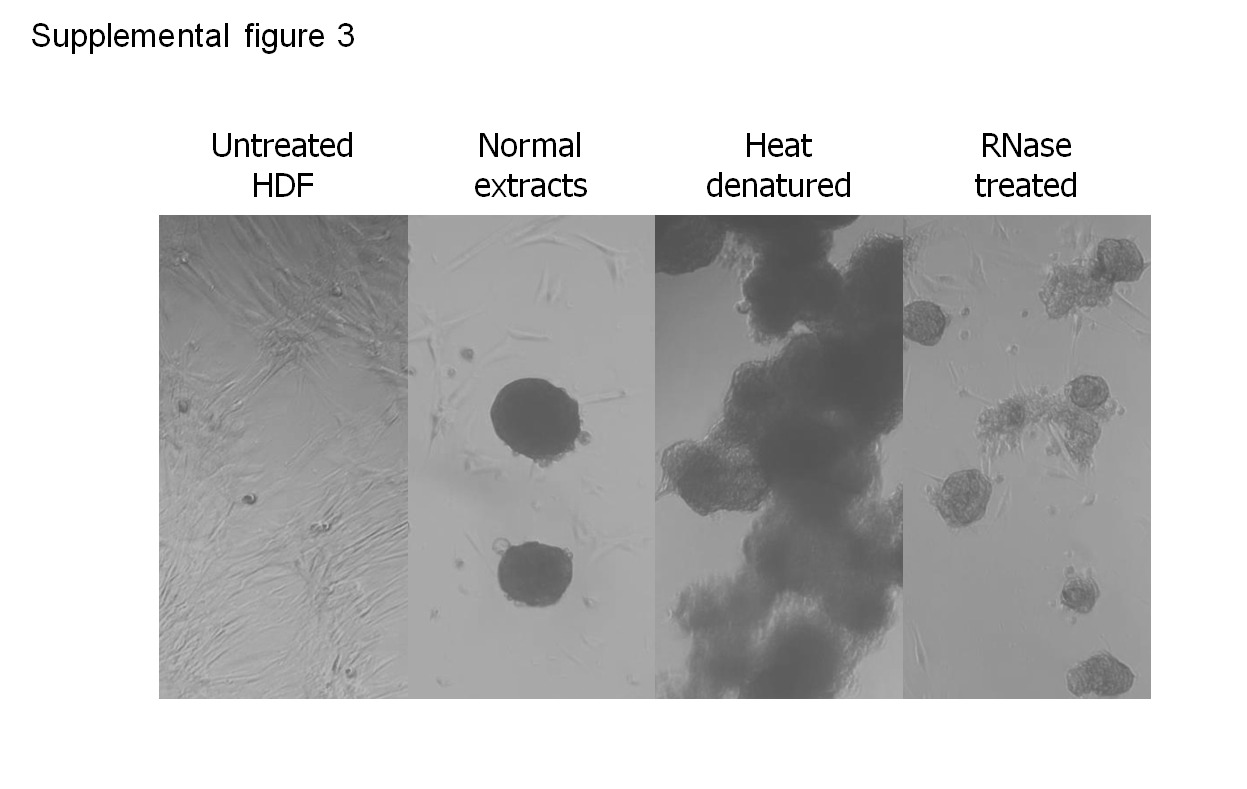

Supplement: Figure S3 — Transfection of HDF with heat- or RNase-treated extracts. When HDF were transfected with either heat-treated NSCL extracts, there was extensive cell death, and when transfected with RNase-treated extracts, they formed smaller spheres. This suggested that mainly protein, and possibly an adjuvant RNA component of the extracts, was responsible for the induction of iNS. (TIF) [file pone.0021801.s003.tif]

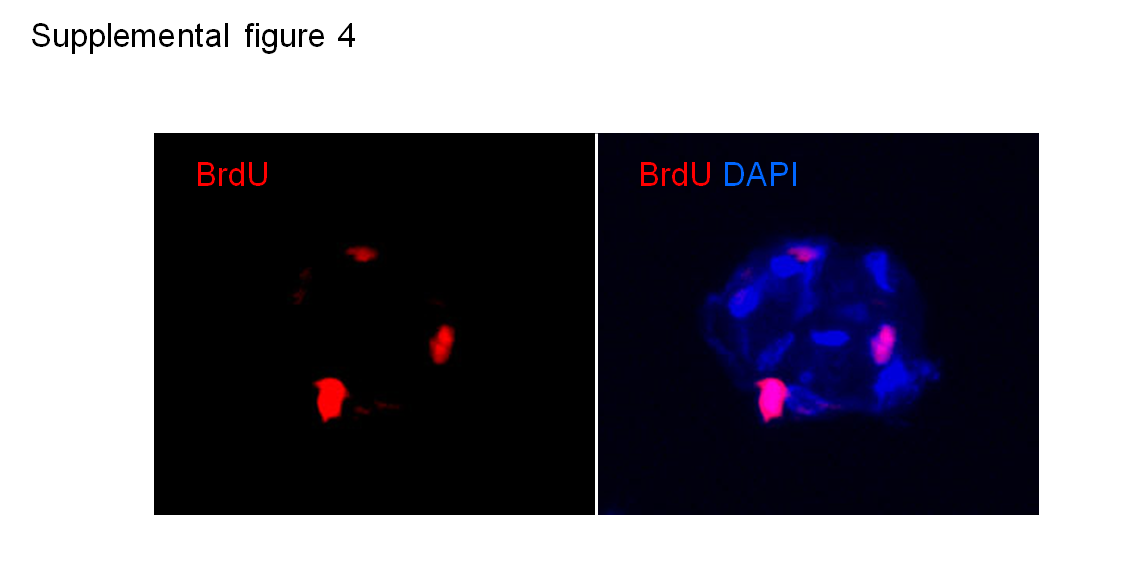

Supplement: Figure S4 — BrdU labeling of iNS. Primary iNS were cultured in neurosphere culture containing BrdU for 3 days. Nuclei were counterstained with diamidino-2-phenylindole (DAPI). A few BrdU-positive cells were detected, indicating proliferation of iNS. (TIF) [file pone.0021801.s004.tif]

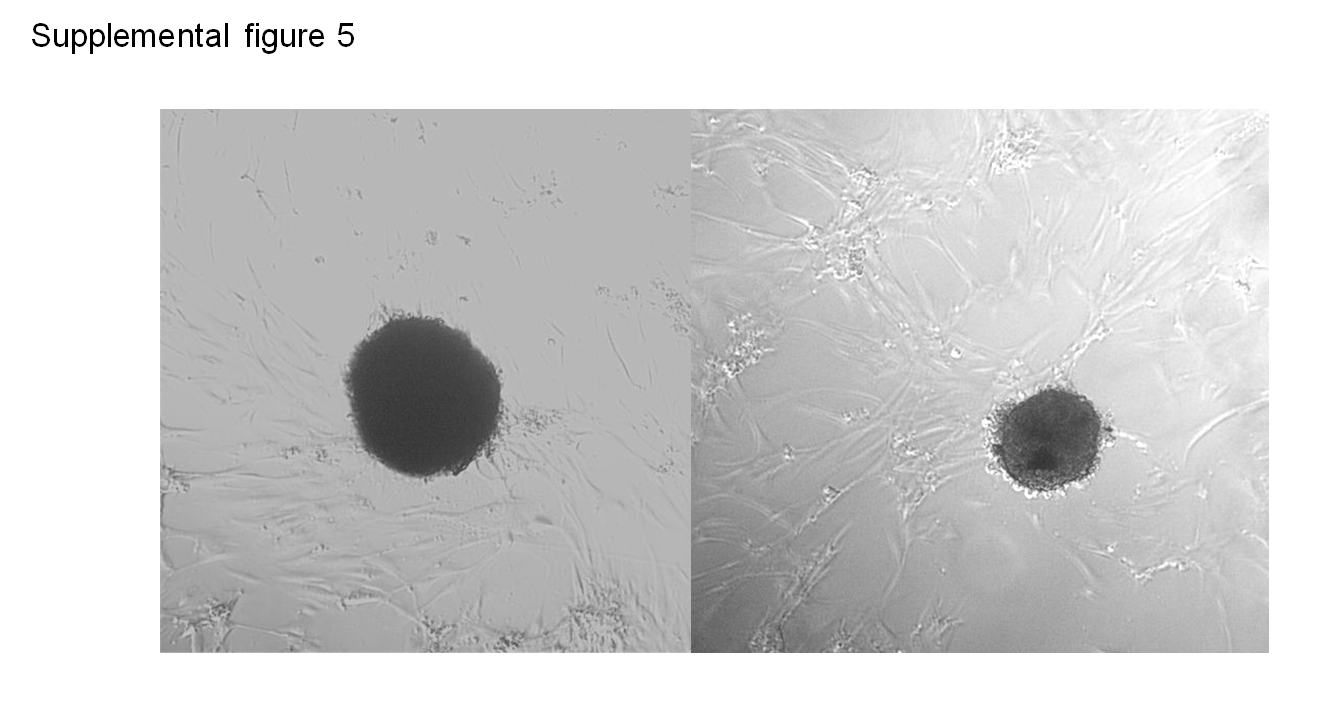

Supplement: Figure S5 — iNS generated by NSCL extracts derived from ReN cells. NSCL extracts derived from ReN cells could induce fibroblasts into neurospheres. (TIF) [file pone.0021801.s005.tif]

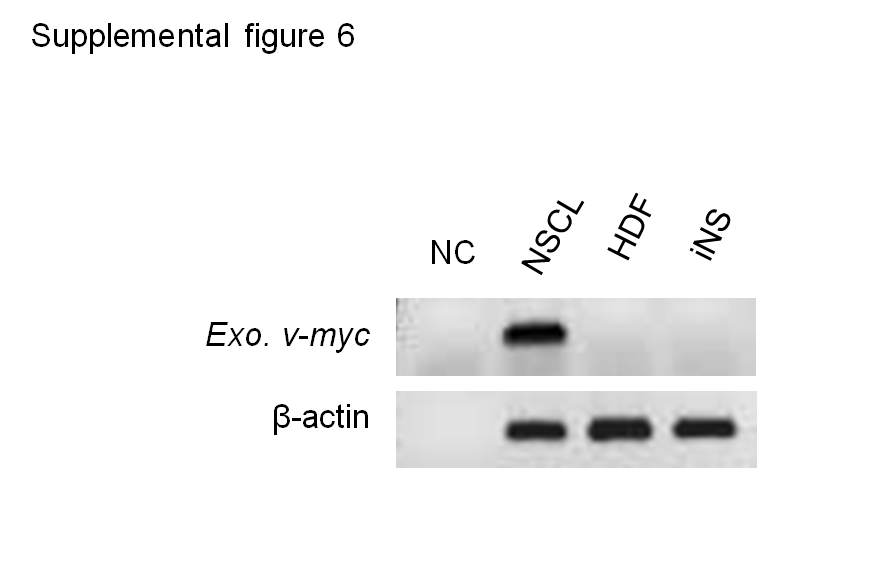

Supplement: Figure S6 — Genomic DNA PCR for v-myc . NSCL were transfected with the v-myc oncogene using a retrovirus. PCR indicated that only NSCL had the v-myc gene. iNS were not contaminated with the gene. (TIF) [file pone.0021801.s006.tif]

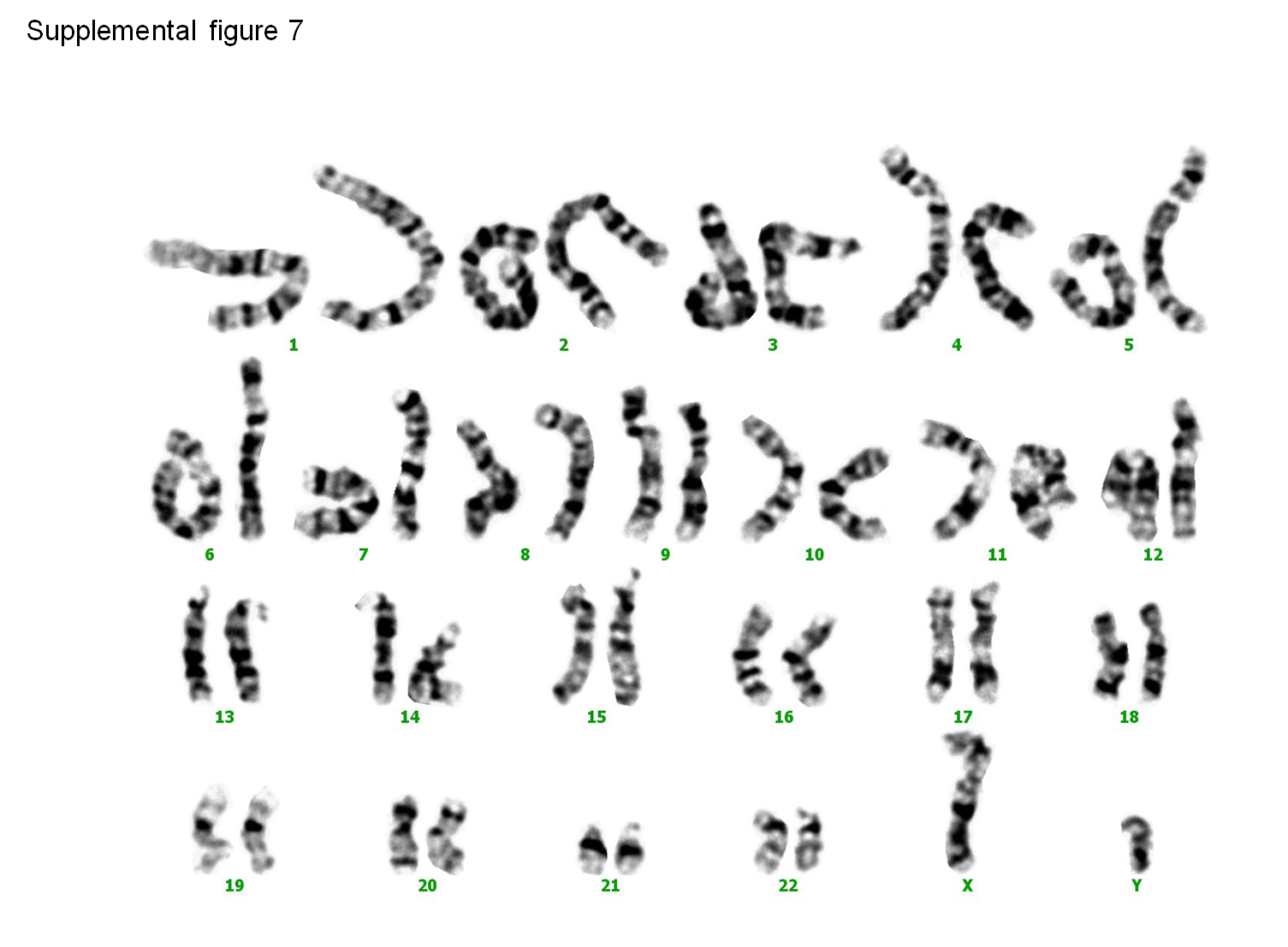

Supplement: Figure S7 — Chromosomal analysis of iNS. iNS have a normal chromosome pattern (46XY). (TIF) [file pone.0021801.s007.tif]

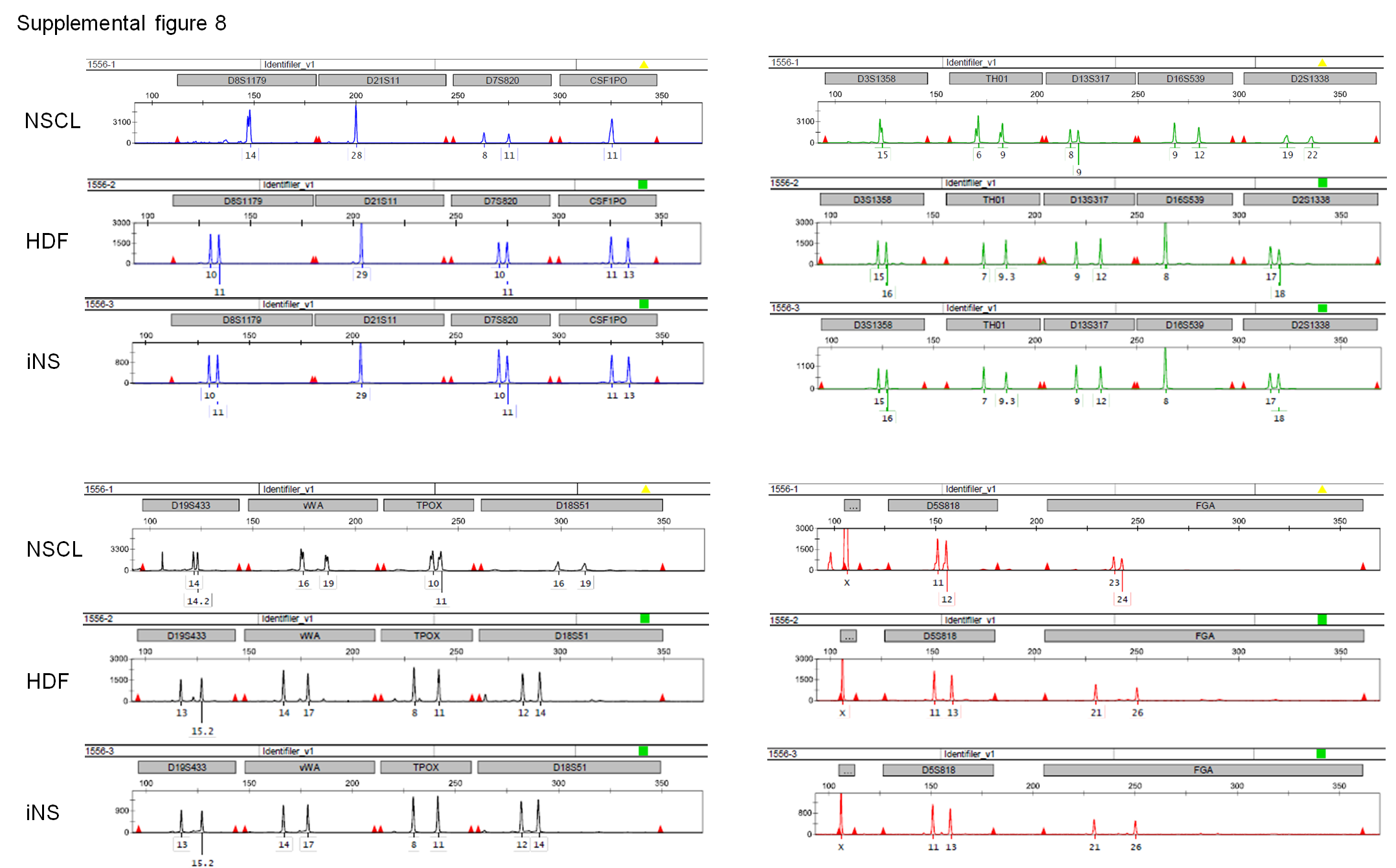

Supplement: Figure S8 — Analysis for short tandem repeats. HDF and iNS have the same patterns of short tandem repeats, whereas the pattern for NSCLs was different. This result confirmed that iNS were derived from HDF, not from NSCLs. (TIF) [file pone.0021801.s008.tif]
