# Supplementary material for: Direct Generation of Neurosphere-Like Cells from Human Dermal Fibroblasts
Source: PLoS One. 2011 Jul 13;6(7):e21801. doi: 10.1371/journal.pone.0021801 (PMC3135606; doi:10.1371/journal.pone.0021801)
Supplement: Table S1 — Primers used in RT-PCR, PCR, and bisulfide sequencing. (DOCX) [file pone.0021801.s009.docx]

**Supplemental table 1. Primers used in RT-PCR, PCR, and bisulfide sequencing**

| Name | Forward (5’🡪 3’) | Reverse (5’🡪 3’) |
| --- | --- | --- |
| SOX2 | CATCACCCACAGCAAATGAC | TTTTTCGTCGCTTGGAGACT |
| Musashi1 | ACCCCCACATTCTCTCACTG | AAACCCAAAACACGAACAGC |
| CD133 | TCAGTGAGAAAGTGGCATCG | TGTTGTGATGGGCTTGTCAT |
| Sox1 | GGGAAAACGGGCAAAATAAT | CCATCTGGGCTTCAAGTGTT |
| Pax6 | TGTGAGTAAAATTCTGGGCAGG | GCAAACACATCTGGATAATGGG |
| Emx2 | GAGAGTTTCCTTTTGCACAACG | ATCTGAGGTCACGTCTATTTCC |
| Dlx2 | CATCCCTTATCTTACCCCCACC | GCTGAGGTCACTG CTAAACTGC |
| Otx2 | GTTCAGAGAGTGGAACAAGTGG | AAGCCTGAGTATAGGTCATGGG |
| En1 | AGCGAGAGAGTTTGTGCAAAGATCC | ACATCCAGATATGGAGACACTTGGC |
| En2 | CGAGTCACAGATCAAGATTTGG | AAAAGGTGAAACCCTAAGCAGC |
| Hoxd3 | TTTGGGTGACTCGCCATAAATCAGC | CAATAAACTGTGGTGAGGACTGTGG |
| MAP2 | CAGGTGGCGGACGTGTGAAAATTGAGAGTG | CACGCTGGATCTGCCTGGGGACTGTG |
| GFAP | GGCCCGCCACTTGCAGGAGTACCAGG | CTTCTGCTCGGGCCCCTCATGAGACG |
| Nestin | AGAGAACCAGGAGCCACTGA | TTCTCTTGTCCCGCAGACTT |
| GAPDH | TCCCCACTGCCAACGTGTCAGTG | ACCCTGTTGCTGTAGCCAAATTCG |
| SRR1 (bisulfite sequencing) | TATTTTTTAGAAAATTGAGTTATTAAGGTA | AATAAAAACTAACCAAACATCTTACTATTA |
| SRR2 (bisulfite sequencing) | TAAAAGAATTTTTTTTAGTTTGGTTAG TA | ACAATACAATTACTATAAACCTCTATACAT |
| v-myc | CCTTTGTTGATTTCGCCAAT | AGTTCTCCTCCTCCTCCTCG |
